# Analysis and enhancement of the energy utilization efficiency of corn stover using strain Lsc-8 in a bioelectrochemical system

**DOI:** 10.1186/s12934-023-02058-6

**Published:** 2023-03-19

**Authors:** Lianbin Cao, Hongmei Sun, Yamei Ma, Mingguo Lu, Mengrui Zhao, Enzhong Li, Ying Liu

**Affiliations:** 1 College of Biological and Food Engineering, Huanghuai University, No. 76 Kaiyuan Road, Zhumadian, 463000 Henan People’s Republic of China; 2 Shaanxi Key Laboratory of Agricultural and Environmental Microbiology, College of Life Sciences, Northwest A&F University, No. 22 Xinong Road, Yangling, 712100 Shaanxi People’s Republic of China

**Keywords:** Microbial fuel cells, Cellulose, Raw corn stover, *Geobacter sulfurreducens* PCA, *Cellulomonas* sp.

## Abstract

The strain Lsc-8 can produce a current density of 33.08 µA cm^−2^ using carboxymethylcellulose (CMC) as a carbon source in a three-electrode configuration. A co-culture system of strain Lsc-8 and *Geobacter sulfurreducens* PCA was used to efficiently convert cellulose into electricity to improve the electricity generation capability of microbial fuel cells (MFCs). The maximum current density achieved by the co-culture with CMC was 559 μA cm^−2^, which was much higher than that of strain Lsc-8 using CMC as the carbon source. The maximum power density reached 492.05 ± 52.63 mW cm^−2^, which is much higher than that previously reported. Interaction mechanism studies showed that strain Lsc-8 had the ability to secrete riboflavin and convert cellulose into acetic acid, which might be the reason for the high electrical production performance of the co-culture system. In addition, to the best of our knowledge, a co-culture or single bacteria system using agricultural straw as the carbon source to generate electricity has not been reported. In this study, the maximum current density of the three-electrode system inoculated with strain Lsc-8 was 14.56 μA cm^−2^ with raw corn stover as the sole carbon source. Raw corn stover as a carbon source was also investigated for use in a co-culture system. The maximum current density achieved by the co-culture was 592 μA cm^−2^. The co-culture system showed a similar electricity generation capability when using raw corn stover and when using CMC. This research shows for the first time that a co-culture or single bacteria system can realize both waste biomass treatment and waste power generation.

**Supplementary Information:**

The online version contains supplementary material available at 10.1186/s12934-023-02058-6.

## Introduction

Global fossil fuel consumption and environmental pollution caused by the burning of fossil fuels are driving research to find carbon–neutral renewable energy [1, 2]. As a unique technology, microbial fuel cells (MFCs) can directly convert the chemical energy of biomass into electricity using exoelectrogens as catalysts and have the potential to become novel systems to produce renewable energy [3, 4]. The substrate is very important for any biological process [5]. The substrate in MFCs systems has a crucial influence on the output power density [5, 6]. Cellulosic biomass, such as corn stover, an ample renewable resource derived from agricultural and industrial byproducts, is attracting increasing attention for use in MFCs systems [7]. Therefore, developing cellulose-fed MFCs has become a hot research topic [8–10].

MFCs employ various undefined mixed cultures as biocatalysts to produce electricity with cellulose [11, 12]. However, for such ambiguous microbial species, the complex metabolites and interaction mechanisms are unknown, which limits the effective optimization of conversion from chemical energy to electrical energy in MFCs systems. Moreover, the biological colony structure of mixed cultures is not stable and is easily affected by environmental changes. Therefore, isolating a single strain that can use a cellulose carbon source and generate electricity at the same time is a key breakthrough to improve the MFCs system. At present, it has been reported that *Enterobacter cloacae* (4.90 ± 0.01 mW m^−2^) [9], *Cellulomonas fimi* (38.70 mW m^−2^) [10] and *Cellulomonas* strain Lsc-8 (9.56 ± 0.37 mW m^−2^) [8] have the ability to use cellulose to produce electricity. However, poor electricity production is the main problem. To enhance the electricity production performance of MFCs. Co-culture systems and ternary culture systems with cellulose as a carbon source have recently been used to improve electricity production in MFCs [13, 14].

On the other hand, agricultural straw with cellulose and lignin as the main components is a relatively rich carbon source. As an agricultural country, China has abundant biomass resources, and straw is the most abundant biomass resource. The direct combustion of straw is the main utilization method of biomass energy in China, which leads to many problems (destroying ecology, polluting the environment and wasting resources). It is essential to find an effective pathway for treating straw. One promising future method is to utilize straw to produce energy through MFCs systems [13, 15]. MFCs can convert organic material directly into electricity. It was proven that corn stover can be used by a mixed culture to produce electricity in a two-compartment MFCs, and the maximum power density is 331 mW m^−2^ [16]. In addition, rumen microbes can use straw to produce electricity in the MFCs system [17]. Studies using straw hydrolysate as the substrate of MFCs have been reported by some researchers [15, 18, 19]. Enzymatic and/or acid hydrolysis can degrade the hemicellulose in agricultural straw to small-molecular sugars, which can be used as an available carbon source for power generation in the MFCs system. Although the utilization efficiency of straw is improved after hydrolysate treatment and the production cost of cellulase is relatively high, the high concentrations of produced acid can also bring about other problems. At present, taking advantage of the electricity production capability of MFCs by using mixed cultures to degrade straw is the main method to solve this problem. It is possible to find new strains with good cellulose degradation capacity or construct new co-culture systems to generate electricity with straw as a carbon source. However, to the best of our knowledge, a single bacterium or co-culture system directly using straw to produce electricity has not been reported.

Strain Lsc-8, a member of the genus *Cellulomonas*, was isolated from rumen content by our group and demonstrated a maximum power density of 9.56 ± 0.37 mW m^−2^ when CMC was used as the carbon source in an MFCs [8]. In this work, the electricity production ability of strain Lsc-8 with CMC or raw corn stover in a three-electrode system was investigated. The amount of electricity generated was low. Thus, we designed a co-culture system including strain Lsc-8 and *Geobacter sulfurreducens* PCA as a biocatalyst for electricity generation with CMC, raw corn stover, hydrolysis residue of corn stover and hydrolysate of corn stover as carbon sources. Compared with the system containing strain Lsc-8, the electricity-producing ability of the co-culture system (strain Lsc-8 and *Geobacter sulfurreducens* PCA) was more than 40 times higher. This research shows that both agricultural biomass treatment and electricity production can be accomplished in MFCs.

## Materials and methods

### Growth media

Strain Lsc-8, belonging to the genus *Cellulomonas*, was isolated from rumen content by our group [8]. The growth medium (FJ) of strain Lsc-8 contained (per liter) 495 mg KH_2_PO_4_, 100 mg NaHCO_3_, 95 mg MgSO_4_·7H_2_O, 930 mg NaCl, 66 mg CaCl_2_·2H_2_O, 495 mg (NH_4_)_2_SO_4_, 653 mg K_2_HPO_4_, 10 mL vitamin solution and 10 mL trace mineral solution. CMC (4.0 g) was used as the carbon source. *Geobacter sulfurreducens* PCA was obtained from Deutsche Sammlung von Mikroorganismen und Zellkulturen GmbH. The growth medium contained (per liter) 1500 mg NH_4_Cl, 600 mg KCl, 100 mg MgCl_2_, 300 mg KH_2_PO4, 100 mg CaCl_2_, 10 mL vitamin solution and 10 mL trace mineral solution, as previously reported [20]. Fumaric acid (4.64 g L^−1^) and sodium acetate (1.64 g L^−1^) were used as an electron acceptor and donor, respectively. All media were adjusted to pH 7.2 before sterilization, and the cells were grown at 30 ± 1 ℃ in a high-purity nitrogen-saturated environment or in air.

### Half-cell experiment

The half-cell experiment was carried out on a potentiostat (Biologic Science Instruments, France) using a three-electrode configuration, as previously reported [13]. A graphite plate (2.60 cm^2^) or carbon fiber cloth (4.50 cm^2^) was used as the electrode. The current density was measured as previously reported [20]. Strain Lsc-8 used carbon fiber cloth as the working electrode, and the co-culture system used a graphite plate as the working electrode. Autoclaved bioreactors were filled with anaerobic medium containing CMC and other carbon sources. Unless otherwise stated in this study, all co-culture experiments were performed anaerobically at 30 ± 1 °C at a constant potential of 0.30 V to maintain highly controlled and consistent conditions.

### MFCs construction

A two-compartment MFCs was used in this study. Each chamber had a working volume of 120 mL and was equipped with a magnetic stirring rod. The co-culture was added to the anode chamber containing anaerobic medium. A carbon rod with a geometric surface area of 6.48 cm^2^ or 3.9 cm^2^ was used as the anode electrode. The cathode chamber was filled with 0.05 M potassium ferricyanide solution, as previously reported [13]. Carbon felt (30 mm × 20 mm × 5 mm) was used as the cathode electrode. The two electrodes were connected by a 1000 Ω resistor. A Keithley instrument (Keithley 2700) monitored the output voltages of the MFCs.

Polarization data were collected by varying the external resistance from 30 kΩ to 100 Ω during steady power generation in each batch experiment. All MFCs were operated anaerobically at 30 ± 1 °C.

### Determination of cellulase activity

The cells in the culture medium were removed by centrifugation (10,000*g*, 15 min at 4 °C). Then, the supernatant of the culture medium was used as the crude cell-free enzyme solution. The 3,5-dinitrosalicylic acid (DNS) colorimetric method was utilized to measure cellulase activity [13]. A 0.5 mL aliquot of crude enzyme was added to wells containing 2.0 mL 50 mM NaAc buffer (CMC-Na, 1% (W/V)). After 15 min of incubation at 50 °C, 2.0 mL of DNS was added to each reaction and incubated at 95 °C for 5 min. Finally, each sample was diluted with water to 10 mL, and the absorbance at 540 nm was measured. One unit is defined as the amount of enzyme that liberates 1 µmol of reducing sugars per min, which is expressed in U/mL.

### Corn stover preparation and hydrolysis

**Fig. 1:**
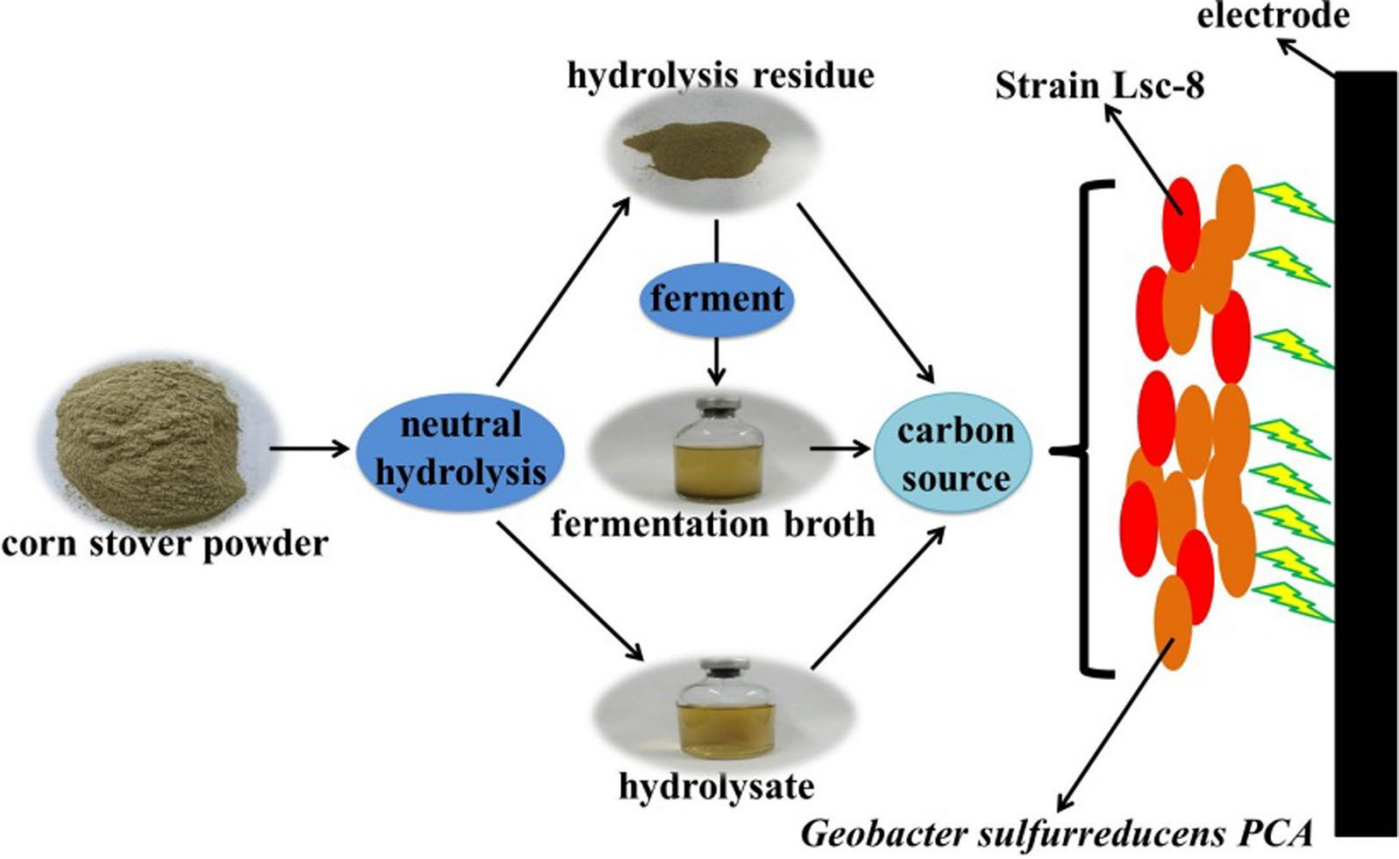
Schematic diagram of raw corn stover powder, hydrolysate of corn stover (medium II) and hydrolysis residue of corn stover (medium III)

Raw corn stover powder (medium I): The corn stover (whole corn except corn ear) (Additional file 1: Fig. S1) collected from Yangling was rinsed with distilled water and further dried at 50 °C. The corn stover was crushed to powder with a size less than 0.5 mm (passed through a 40 mesh screen) (Fig. 1 and Additional file 1: Fig. S1). Then, the corn stover powder was used directly as the carbon source. FJ medium was used as the growth medium. Corn stover powder (4 g L^−1^) was used as the carbon source. Sterilization was performed at 121 °C for 20 min.

Supernatant of medium I: After the raw corn stover powder was sterilized at 121 °C for 20 min, the supernatant was collected after centrifugation (12,000 rpm).

Hydrolysate of corn stover (medium II): A total of 4 g corn stover powder was treated with high-pressure steam (121 °C) for 2 h in 1000 mL water (neutral hydrolysis). After cooling, filtration was performed to remove solids, and the hydrolysate solution was collected. Before being used in tests, the hydrolysate solution was supplemented with (per liter) 495 mg KH_2_PO_4_, 1000 mg NaHCO_3_, 930 mg NaCl, 95 mg MgSO_4_·7H_2_O, 495 mg (NH_4_)_2_SO_4_, 66 mg CaCl_2_·2H_2_O, 653 mg K_2_HPO_4_, 10 mL vitamin solution and 10 mL trace mineral solution (same as in the FJ medium). Sterilization was performed at 121 °C for 20 min.

Hydrolysis residue of corn stover (medium III): A total of 4 g corn stover powder was treated with high-pressure steam (121 °C) for 2 h in 1000 mL water (neutral hydrolysis). After cooling, the solids were filtered and washed with distilled water to collect the hydrolysis residue. Then, the hydrolysis residue was used as the carbon source. FJ medium was used as the growth medium. Hydrolysis residue (4 g L^−1^) was used as the carbon source. Oxygen was used as an electron acceptor. Finally, the growth medium with hydrolysis residue was sterilized at 121 °C for 20 min.

Fermentation broth of medium III: Strain Lsc-8 of the co-culture system was used to ferment medium III, and then the fermentation broth of medium III was used as the carbon source.

### Chemical oxygen demand and coulombic efficiency

Chemical oxygen demand (COD) was measured according to standard methods [21].

The Coulombic efficiency (CE) for an MFCs run in fed-batch mode, evaluated over a period of time t, is calculated as

1 $$CE=\frac{M{\int }_{0}^{t}Idt}{FbV\Delta COD}$$

where M is the molecular weight of oxygen (32), F is Faraday’s constant, b = 4 indicates the number of electrons exchanged per mole of oxygen, V is the volume of the liquid in the anode chamber, and ∆COD is the change in COD over time t.

### Analysis of products in solution

The reducing sugars in the solution were detected by the DNS method [13]. The anthrone sulfate method was used to determine the total soluble sugar content of the solution [22]. Moreover, high-performance liquid chromatography (HPLC, Agilent 1260) was used to determine the acetate concentration [23].

## Results and discussion

### Electricity generation performance of strain Lsc-8

**Fig. 2:**
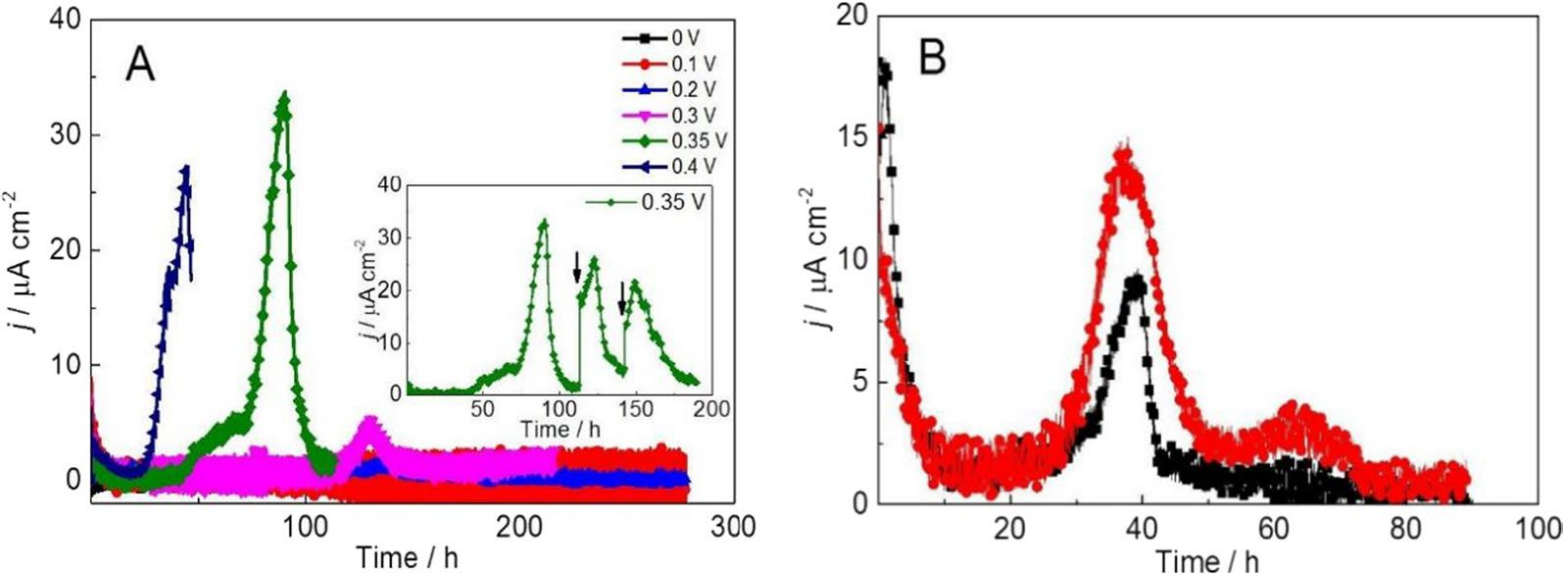
**A** Chronoamperometric curves of three-electrode systems inoculated with strain Lsc-8 with CMC as the carbon source at different constant potentials; the inset shows chronoamperometric curves of three-electrode systems inoculated with strain Lsc-8 with CMC as the carbon source at a constant potential of 0.35 V; arrows indicate the replacement of half of the solution in the reactor with new CMC medium. **B** Chronoamperometric curves of three-electrode systems inoculated with strain Lsc-8 with medium I as the carbon source at a constant potential of 0.35 V

The output current densities of the three-electrode systems inoculated with strain Lsc-8 with CMC as the carbon source were different at different constant potentials. No current densities were detected at constant potentials of 0 V and 0.1 V (Fig. 2). Current densities of 1.28 µA cm^−2^ and 4.30 µA cm^−2^ were detected at constant potentials of 0.2 V and 0.3 V, respectively (Fig. 2). When the constant potential was increased to 0.35 V, the output current density increased rapidly to 33.08 µA cm^−2^ (Fig. 2). However, the output current density did not increase as the constant potential continued to increase. These data showed that strain Lsc-8 could use CMC as an electron donor to produce electricity in a three-electrode system and that the magnitude of the potential could affect the output current density. The energy per electron released during the electrochemical reaction directly depends on the potential. The potential drives electrochemical reactions, and within a certain range, with increasing potential, the reaction rate should increase [24]. Therefore, the output current density increased as the potential increased within a certain range (Fig. 2). This relationship between potential and current density produced by microorganisms has also been observed in some reports [25–27]. In addition, when we used fumarate, glycerin, glucose and acetate as electron donors, the current density ranged from 16.32 to 33.39 μA cm^−2^ (Additional file 1: Fig. S2).

Based on the maximum current density of strain Lsc-8, a constant potential of 0.35 V was adopted as the optimal potential in the bioreactor using CMC as the carbon source (Fig. 2). Thus, the continuous electricity generation capability of the three-electrode system inoculated with strain Lsc-8 with CMC as the carbon source under a constant potential of 0.35 V was studied (Fig. 2 inset). When the current density dropped below 5 µA cm^−2^, half of the solution in the bioreactor was replaced with new CMC medium. However, after replacing the new medium, the maximum current density of 26.28 µA cm^−2^ was lower than the maximum of 33.08 µA cm^−2^ obtained for the initial batch. The output current density (21.41 µA cm^−2^) was further reduced in the third batch. This may be because the accumulation of dead cells and metabolic waste on the carbon fiber cloth working electrode affects electron transfer from strain Lsc-8 to the working electrode with the operation of the reactor.

Furthermore, the electricity production ability of strain Lsc-8 with raw corn stover in a three-electrode system at a constant potential of 0.35 V was studied (Fig. 2B). Strain Lsc-8 could produce a current density of 14.56 μA cm^−2^ from raw corn stover in the three-electrode system. The electricity production ability of strain Lsc-8 with raw corn stover as the carbon source was also studied in an MFCs system. The maximum output voltage was 44.51 ± 1.38 mV (Additional file 1: Fig. S3), and the maximum output power density was 6.19 ± 0.59 mW m^−2^ (Additional file 1: Fig. S4), which was similar to that obtained with strain Lsc-8 and glucose in an MFCs [8]. These results showed that strain Lsc-8 might be capable of generating electricity with raw corn stover as the carbon source. To date, pure cultures of electrochemically active bacteria that can use CMC as an electron donor to produce electricity in MFCs have been reported by some researchers [8–10]. However, pure cultures of electrochemically active bacteria using CMC to generate electricity in a three-electrode system and using straw to generate electricity have not been reported.

In this study, strain Lsc-8 could use CMC to produce electricity in a three-electrode system, but the electricity production was very low. Previous reports found the same results [8–10]. *Cellulomonas* sp. can utilize cellulose to produce acetic acid [28, 29] and *G. sulfurreducens* PCA can use acetate to produce electricity. Therefore, building a co-culture system to improve the electricity production of the entire reaction system was the focus of the next study.

### Co-culture system generating electricity with CMC

**Fig. 3:**
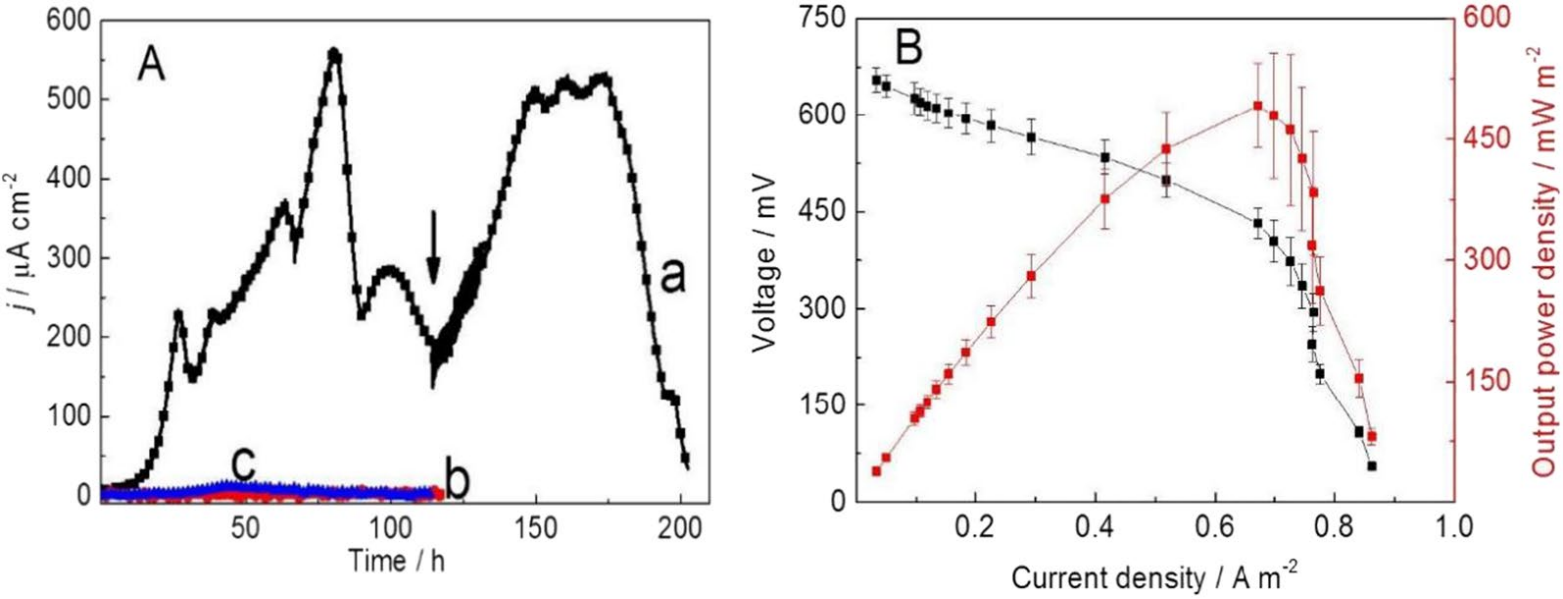
**A** Chronoamperometric curves of three-electrode systems inoculated with the co-culture with CMC (curve a) and without a carbon source (curve b) or inoculated *with Geobacter sulfurreducens* PCA with CMC (curve c) at a constant potential of 0.3 V. The arrow indicates the replacement of the solution in the reactor with CMC. **B** Voltage and output power density *vs.* current density obtained by varying the external circuit resistance; co-culture with CMC as the carbon source

Chronoamperometric curves of the three-electrode systems inoculated with the co-culture (strain Lsc-8 and *G. sulfurreducens* PCA) with CMC as the carbon source are shown in Fig. 3A. The current density was 559 μA cm^−2^ at 80 h (Fig. 3A curve a). When the current density was reduced, which may have been due to CMC depletion or metabolite accumulation, the solution in the bioreactor was replaced with new CMC medium. After replacing the medium, the current density recovered to 531 μA cm^−2^, which was comparable to that of the initial batch. Cyclic voltammetry curves were measured at the highest and lowest points of current density (Additional file 1: Fig. S6). The results showed an obvious oxygen reduction peak at the low point of current density (Fig. S6 curve b). Correspondingly, two controlled experiments showed that the co-culture system could not produce electricity without a carbon source in the medium (Fig. 3A curve b). More importantly, *G. sulfurreducens* PCA could use acetate to produce a current density of 530 μA cm^−2^ (Additional file 1: Fig. S5) but could not use CMC to generate electricity (Fig. 3A curve c). These results indicated that the co-culture system could utilize CMC to generate electricity and that the maximum current density (559 μA cm^−2^) of the co-culture system was far higher than that of strain Lsc-8 (33.08 µA cm^−2^). Strain Lsc-8 can utilize cellulose to produce acetic acid [28, 29], which can be used by *G. sulfurreducens* PCA to generate electricity. In addition, glucose was studied as an electron donor, and the results are shown in Additional file 1: Fig. S7. The co-culture system could produce a current density of 327 µA cm^−2^ from glucose.

The electricity generation performance of the co-culture system (strain Lsc-8 and *G. sulfurreducens* PCA) with CMC was further investigated via an MFCs system (Fig. 3B and Additional file 1: Fig. S8). The maximum output voltage was as high as 456 mV (Additional file 1: Fig. S8). The maximum current density reached 1200 mA m^−2^, with an external resistance of 1 kΩ. The maximum power density of the MFCs co-culture system with CMC as the carbon source was 492.05 ± 52.63 mW m^−2^ (Fig. 3B), which was more than three times higher than that (143 mW m^−2^) of a previously reported co-culture system (*G. sulfurreducens* and *Clostridium cellulolyticum*) in an MFCs [14]. Strain Lsc-8 has the ability to secrete riboflavin, which can improve the electrical performance of exoelectrogens [8]. This might be the reason for the high electrical production performance of the co-culture system in this study. Although a co-culture system using CMC as a carbon source has been reported, building a new co-culture system with high power generation using CMC as a carbon source is crucial.

The cellulase activity of strain Lsc-8 was also determined. When the strain was fed with CMC, the cellulase activity was 0.20 ± 0.0071 U mL^−1^ (Additional file 1: Table S1). In view of the ability of strain Lsc-8 to degrade cellulose, can raw corn stover be considered a carbon source for the co-culture system? To address this question, a co-culture system using raw corn stover as the carbon source to generate electricity was studied next.

### Electricity generation in a co-culture system with medium I

**Fig. 4:**
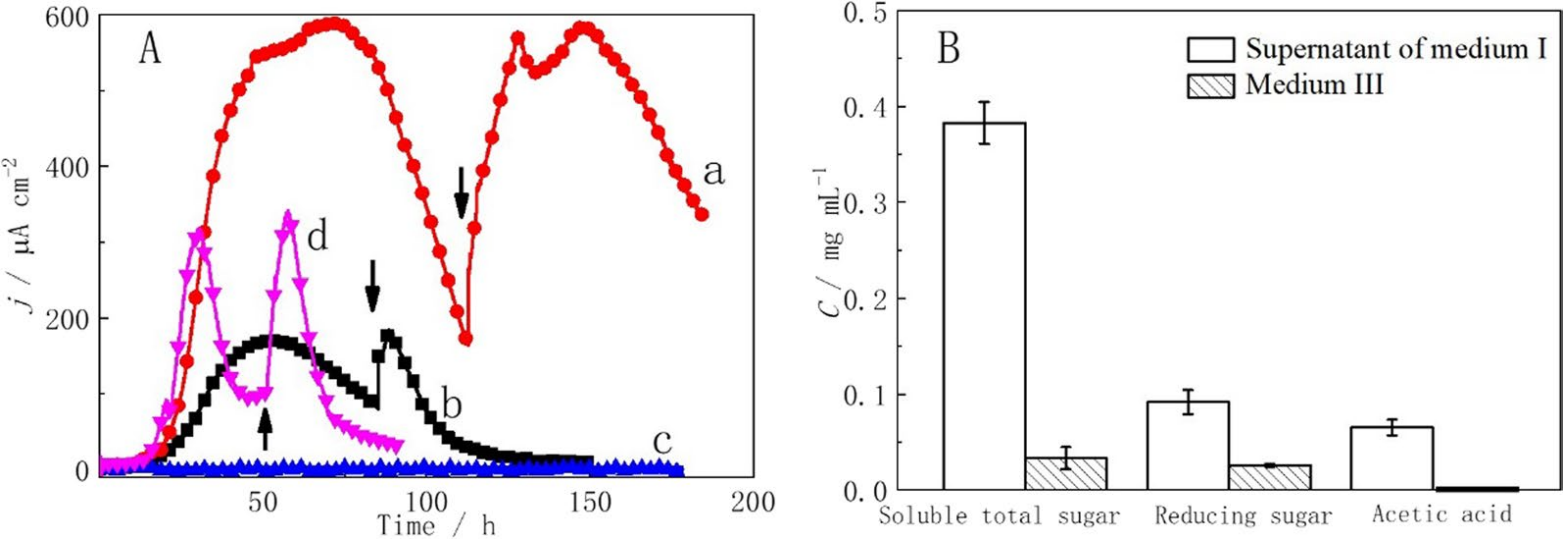
**A** Chronoamperometric curves of the three-electrode system inoculated with co-culture with medium I (curve a), *Geobacter sulfurreducens* PCA with medium I (curve b), co-culture without a carbon source (curve c) and co-culture with supernatant of medium I (curve d) at a constant potential of 0.3 V. The arrows indicate the replacement of the solution in the reactor with the same carbon source. **B** Concentrations of total soluble sugars, reducing sugars and acetic acid in the supernatant of medium I and medium III

The co-culture system with medium I as the carbon source produced a maximum current density of 592 μA cm^−2^ in the three-electrode system (Fig. 4A curve a). When the current decreased, the solution was replaced with new medium containing the same carbon source, and the current recovered to 588 μA cm^−2^ in 10 h. Accordingly, *G. sulfurreducens* PCA with medium I and a co-culture system without a carbon source were used as the control experiments. When there was no carbon source in solution, the co-culture system hardly produced electricity (Fig. 4A curve c). These data illustrated that the co-culture system could utilize raw corn stover to generate electricity. Moreover, the co-culture system showed a similar electricity generation capability when using raw corn stover and when using CMC. The electricity generation performance of this co-culture system with raw corn stover as the carbon source in an MFC is equivalent to or slightly higher than that of hydrolysate used by mixed bacteria [15, 16, 18, 30–32]. However, the CE of MFCs with raw corn stover as the carbon source was 26.59%, which was slightly higher than that (22.42%) with CMC as the carbon source. This may be because some acetate in raw corn stover can be directly used by *G. sulfurreducens* PCA to generate electricity, while CMC needs to be degraded into acetate before it can be used by *G. sulfurreducens* PCA to generate electricity. It has been reported that the CE of MFCs with monosaccharides or polysaccharides as the carbon source was lower than that of MFCs with acetate and propionate because fermentable monosaccharides or polysaccharides support nonelectrogenic microorganism metabolism [15, 33].

Unexpectedly, *G. sulfurreducens* PCA could produce a maximum current density of 171 μA cm^−2^ when fed with medium I (Fig. 4A curve b). When the solution was replaced with new medium, the current recovered to 179 μA cm^−2^ in 5 h, which was comparable to that of the initial batch (Fig. 4A curve b). Some small-molecule soluble substances in the corn stover [34] dissolved during the 121 °C sterilization process of medium I. In the supernatant of medium I, an acetic acid concentration of 0.065 ± 0.0083 mg mL^−1^ was detected (Fig. 4B). This acetic acid could be utilized by *G. sulfurreducens* PCA to generate electricity. However, the maximum current density (179 μA cm^−2^) of *G. sulfurreducens* PCA was much lower than that (592 μA cm^−2^) of the co-culture system with medium I (Fig. 4A curves a and b). In addition, in the co-culture system with the supernatant of medium I, the maximum current density was 313 μA cm^−2^, which was also lower than that (592 μA cm^−2^) of the co-culture system with medium I but higher than that (179 μA cm^−2^) of *G. sulfurreducens* PCA with medium I (Fig. 4A curves a, b and d). After medium I was sterilized at 121 °C, 0.092 ± 0.013 mg mL^−1^ reducing sugars and 0.38 ± 0.022 mg mL^−1^ total soluble sugars were detected in the supernatant of medium I (Fig. 4B). These data showed that except for some small-molecule soluble substances, many soluble sugars in corn stover dissolved during the 121 °C sterilization process. These soluble sugars could be utilized by strain Lsc-8 to produce more acetic acid, which could be used by *G. sulfurreducens* PCA to generate electricity. *Cellulomonas* sp. has the ability to degrade straw [35, 36]. Under the action of enzymes, in addition to soluble sugars, polysaccharides (such as cellulose, hemicellulose and starch) in corn stover will be degraded by strain Lsc-8 to produce acetic acid [28, 29]. Therefore, compared to the supernatant of medium I, more acetic acid could be produced by strain Lsc-8 with medium I.

### Electricity generation in co-cultures with medium II and medium III

**Fig. 5:**
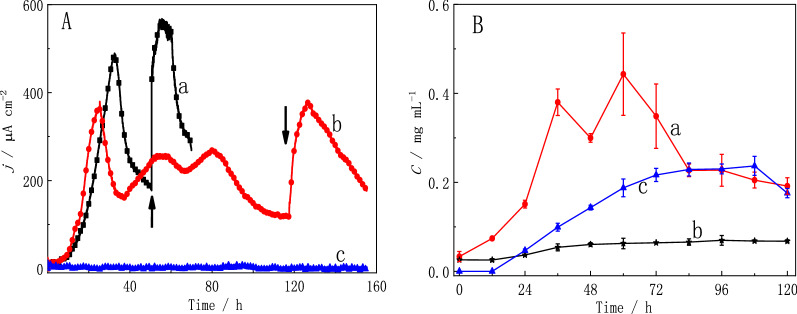
**A** Chronoamperometric curves of three-electrode systems inoculated with the co-culture with medium II (curve a) and fermentation broth of medium III (curve b) or *G. sulfurreducens* PCA with medium III (curve c) as the carbon sources. Arrows indicate the replacement of the solution in the reactor with the same carbon source. **B** Line chart showing the change in total soluble sugar (curve a), reducing sugar (curve b) and acetic acid (curve c) concentrations over time in the solution with the fermentation broth of medium III

In the co-culture system with medium II, a maximum current density of 490 μA cm^−2^ was detected in the three-electrode system (Fig. 5A curve a). After replacing the medium with new medium, the current density recovered to 556 μA cm^−2^ in 10 h, which was lower than the current density (592 μA cm^−2^) of the co-culture system with raw corn stover as the carbon source. After the raw corn stover was treated with neutral hydrolysis, some soluble sugars and soluble small-molecule substances in the corn stover dissolved, but undissolved carbon sources (such as cellulose, hemicellulose and starch) in the corn stover could not be dissolved. These undissolved carbon sources might be used by the co-culture system to produce electricity. In addition, we measured the COD of the anode chamber solution at the beginning and end of the three-electrode system operation. The results showed that the COD removal rate in the three-electrode system was 41.13 ± 4.76%. The COD removal rate was lower than that (68%) in previous reports of corn stover biomass [32]. This may be because the degradation performance of organic matter of the binary strains is lower than that of the mixed strains.

Therefore, to better verify the ability of strain Lsc-8 to utilize undissolved carbon sources in corn stover, neutral hydrolysis was used to treat raw corn stover to remove the soluble carbon sources (Fig. 1). After medium III was sterilized at 121 °C, the concentrations of reducing sugars and total soluble sugars in solution were detected (Fig. 4B). The concentrations of reducing sugars (0.026 ± 0.0019 mg mL^−1^) and total soluble sugars (0.033 ± 0.011 mg mL^−1^) in the supernatant of medium III were much lower than those (0.092 ± 0.013 mg mL^−1^ and 0.38 ± 0.022 mg mL^−1^) in the supernatant of medium I (Fig. 4B). These data showed that neutral hydrolysis removed most of the soluble carbon sources in the raw corn stover. Thus, medium III was used for subsequent tests. With medium III, a maximum current density of 293 μA cm^−2^ was detected in 42 h (Additional file 1: Fig. S11). However, this maximum current density of 293 μA cm^−2^ was lower than the value of 592 μA cm^−2^ obtained for the co-culture system with raw corn stover as the electron donor. Due to the removal of soluble carbon sources in the corn stover, the available carbon sources in medium III were reduced. Therefore, strain Lsc-8 could not produce enough acetic acid for *G. sulfurreducens* PCA to utilize. Accordingly, a system containing *G. sulfurreducens* PCA with medium III was established as the control experiment. *G. sulfurreducens* PCA hardly produces electricity with medium III (Fig. 5A curve c). In the supernatant of medium III, acetic acid was not detected (Fig. 4B). These data showed that the co-culture system could use the hydrolysis residue of corn stover to produce electricity.

Strain Lsc-8 in the co-culture system was used to ferment medium III first, and then the fermentation broth was used as the carbon source for the co-culture system. Figure 5B shows the change in the concentrations of reducing sugars and total soluble sugars with fermentation time in medium III. The concentration of total soluble sugars increased from 0.033 ± 0.011 mg mL^−1^ to 0.44 ± 0.093 mg mL^−1^ in the fermentation broth at 60 h (Fig. 5B curve a), while the concentration of reducing sugars gradually increased at 84 h (Fig. 5B curve b). These data indicated that strain Lsc-8 could degrade the undissolved carbon sources of medium III to produce some sugars. This also illustrated that strain Lsc-8 had the ability to degrade corn stover. However, the concentration of total soluble sugars gradually decreased after 60 h (Fig. 5B curve a). The concentration of soluble sugars produced by strain Lsc-8 decreased with the reduction in available carbon sources in medium III, but the soluble sugars in solution were used all the time by strain Lsc-8. Therefore, the concentration of soluble sugars in solution began to decrease after 60 h.

Then, the fermentation broth obtained at 60 h was used as the carbon source for the next tests. Moreover, the change in the concentration of acetic acid in solution in medium III with fermentation time was detected (Fig. 5B curve c). The concentration of acetic acid increased from 0 mg mL^−1^ to 0.24 ± 0.021 mg mL^−1^ in 108 h. These data showed that strain Lsc-8 could use the hydrolysis residue of corn stover to produce acetic acid, which can be used by *G. sulfurreducens* PCA to produce electricity. In the three-electrode co-culture system with the fermentation broth of medium III as the electron donor, a maximum current density of 378 μA cm^−2^ was detected (Fig. 5A curve b). After replacing the medium, the current density recovered to 382 μA cm^−2^, which was comparable to that of the initial batch (Fig. 5A curve b). The maximum current density of 378 μA cm^−2^ was also lower than the value of 592 μA cm^−2^ obtained for the co-culture system with raw corn stover as the electron donor but was higher than the value of 293 μA cm^−2^ obtained for the co-culture system with medium III. However, after adding a carbon source to the medium, the current density increased to 541 μA cm^−2^ (Additional file 1: Fig. S12). Moreover, a shorter adaptation time (24.72 ± 1.85 h) was required for the co-culture system with the fermentation broth of medium III than for the co-culture system with medium III (37.28 ± 4.54 h) (Additional file 1: Fig. S11). The lack of soluble carbon sources made medium III more difficult for strain Lsc-8 to use. Strain Lsc-8 needed time to degrade the hydrolysis residue of corn stover to produce soluble sugars and acetic acid (Fig. 5B), so strain Lsc-8 could not provide enough carbon sources for *G. sulfurreducens* PCA in time. During the fermentation process, strain Lsc-8 could utilize the hydrolysis residue of corn stover to produce soluble sugars, which were easily used by the co-culture system. This provided *G. sulfurreducens* PCA with more carbon sources to produce electricity in a short time, but these carbon sources were not sufficient for *G. sulfurreducens* PCA.

Furthermore, the electricity generation performance of the co-culture system with the fermentation broth of medium III was further investigated in an MFCs system (Additional file 1: Fig. S13). The output voltages of the MFCs were as high as 260 mV (Additional file 1: Fig. S13A). With an external resistance of 1 kΩ, the maximum current density reached 684 mA m^−2^. The maximum power density was 219.90 ± 31.47 mW m^−2^ (Additional file 1: Fig. S13B). Mixed cultures using straw or straw hydrolysate as the carbon source to generate electricity have been reported by some researchers[17–19]. However, a co-culture system using straw or straw hydrolysate as the carbon source to generate electricity has not been reported.

## Conclusion

Strain Lsc-8 could produce current densities of 33.08 μA cm^−2^ and 14.56 μA cm^−2^ using CMC and raw corn stover as carbon sources in a three-electrode system. Strain Lsc-8 can efficiently convert cellulose into acetate, which can be used by *G. sulfurreducens* PCA to produce electricity. Thus, a defined co-culture system of strain Lsc-8 and *G. sulfurreducens* PCA was constructed to improve electricity generation. The maximum current density of the three-electrode co-culture system with CMC was 559 μA cm^−2^, which was higher than that of strain Lsc-8. The maximum power density of the MFCs co-culture system with CMC was 492.05 ± 52.63 mW m^−2^, which was much higher than that of a previously reported co-culture system (*G. sulfurreducens* and *C. cellulolyticum*) using CMC as the carbon source. In addition, the maximum current densities of the three-electrode co-culture systems with raw corn stover, corn stover hydrolysate and hydrolysis residue of corn stover as the carbon sources were 592 μA cm^−2^, 490 µA cm^−2^ and 382 µA cm^−2^, respectively. The co-culture system showed similar electricity generation capability when using raw corn stover and when using CMC. To date, a co-culture system using straw or straw hydrolysis residue as the carbon source to generate electricity has not been reported. The changes in the concentrations of reducing sugars, total soluble sugars and acetic acid with fermentation time in medium III were also detected. Strain Lsc-8 demonstrated the ability to degrade corn stover to produce acetic acid. This research shows the realization of both waste biomass treatment and electricity generation.

## Supplementary Information

**Additional file 1: Fig. S1.** Corn stover (whole corn except corn ear) was crushed into less than 0.5 mm powder (passed through a 40 mesh screen). **Fig. S2.** Chronoamperometric curves of the three-electrode system inoculated with strain Lsc-8 with different carbon sources at a constant potential of 0.35 V. **Fig. S3.** The output voltages of the MFCs inoculated with strain Lsc-8 with raw corn stover as the carbon source; curves a, b and c denote three replications. **Fig. S4.** Curves of the output voltage and power density as a function of current density in MFCs inoculated with strain Lsc-8 with corn stover as the carbon source. **Fig. S5.** Chronoamperometric curves of the three-electrode system inoculated with *G. sulfurreducens* PCA with acetate as the carbon source at a constant potential of 0.3 V. **Fig. S6.** Cyclic voltammograms of the three-electrode inoculated co-culture system with CMC as the carbon source: (a) when the output current density reached its highest value, (b) when the output current density dropped to zero, (c) at the start of operation (the control). **Fig. S7.** Chronoamperometric curves of the three-electrode inoculated co-culture system (curves a and b denote two replications) or *G. sulfurreducens* PCA (curve c) with glucose as the carbon source at a constant potential of 0.3 V. **Fig. S8.** The output voltages of the MFCs-inoculated co-culture system with CMC as the carbon source. Arrows show replacement of the medium in the MFCs with new CMC medium. **Fig. S9.** The standard curve of OD_540 nm_ versus glucose concentration (reducing sugar assay). **Fig. S10.** The standard curve of OD_620 nm_ versus glucose concentration (soluble total sugar assay). **Fig. S11.** Chronoamperometric curves of the three-electrode inoculated co-culture system with medium III (curves d, e and f denote three replications) and fermentation broth of medium III (curves a, b and c denote three replications) as the carbon source. **Fig. S12.** Chronoamperometric curves of the three-electrode inoculated co-culture system with fermentation broth of medium III as the carbon source. Arrows indicate the addition of acetate. **Fig. S13.** (A) The output voltages of the MFCs-inoculated co-culture system with fermentation broth of medium III as the carbon source. Arrows show replacement of the medium in the MFCs with the same carbon source. (B) Curves of the output voltage and power density as a function of current density in the MFCs-inoculated co-culture system with fermentation broth of medium III as the carbon source. **Fig. S14.** The standard curve of peak area versus acetic acid concentration. **Table S1.** The cellulase activity of strain Lsc-8.

## Data Availability

The data of this study can be shared openly. All data can be obtained from the corresponding author or first author.
