# Supplementary material for: Analysis and enhancement of the energy utilization efficiency of corn stover using strain Lsc-8 in a bioelectrochemical system
Source: Microb Cell Fact. 2023 Mar 19;22:54. doi: 10.1186/s12934-023-02058-6 (PMC10024844; doi:10.1186/s12934-023-02058-6)
Supplement: Supplementary file 1 — Additional file 1: Fig. S1. Corn stover (whole corn except corn ear) was crushed into less than 0.5 mm powder (passed through a 40 mesh screen). Fig. S2. Chronoamperometric curves of the three-electrode system inoculated with strain Lsc-8 with different carbon sources at a constant potential of 0.35 V. Fig. S3. The output voltages of the MFCs inoculated with strain Lsc-8 with raw corn stover as the carbon source; curves a, b and c denote three replications. Fig. S4. Curves of the output voltage and power density as a function of current density in MFCs inoculated with strain Lsc-8 with corn stover as the carbon source. Fig. S5. Chronoamperometric curves of the three-electrode system inoculated with G. sulfurreducens PCA with acetate as the carbon source at a constant potential of 0.3 V. Fig. S6. Cyclic voltammograms of the three-electrode inoculated co-culture system with CMC as the carbon source: (a) when the output current density reached its highest value, (b) when the output current density dropped to zero, (c) at the start of operation (the control). Fig. S7. Chronoamperometric curves of the three-electrode inoculated co-culture system (curves a and b denote two replications) or G. sulfurreducens PCA (curve c) with glucose as the carbon source at a constant potential of 0.3 V. Fig. S8. The output voltages of the MFCs-inoculated co-culture system with CMC as the carbon source. Arrows show replacement of the medium in the MFCs with new CMC medium. Fig. S9. The standard curve of OD540 nm versus glucose concentration (reducing sugar assay). Fig. S10. The standard curve of OD620 nm versus glucose concentration (soluble total sugar assay). Fig. S11. Chronoamperometric curves of the three-electrode inoculated co-culture system with medium III (curves d, e and f denote three replications) and fermentation broth of medium III (curves a, b and c denote three replications) as the carbon source. Fig. S12. Chronoamperometric curves of the three-electrode inoculated co-cultu [file 12934_2023_2058_MOESM1_ESM.docx]

**Analysis and Enhancement of the Energy Utilization Efficiency of Corn Stover using Strain Lsc-8 in a Bioelectrochemical System**

Lianbin Cao^a^, Hongmei Sun^a^, Yamei Ma^b^, Mingguo Lu^a^, Mengrui Zhao ^a^, Enzhong Li^a^, Ying Liu^b*^

^a^ College of Biological and Food Engineering, Huanghuai University, No. 76 Kaiyuan Road, Zhumadian, Henan Province, PR China 463000

^b^ Shaanxi Key Laboratory of Agricultural and Environmental Microbiology, College of Life Sciences, Northwest A&F University, No. 22 Xinong Road, Yangling, Shaanxi Province, PR China 712100

* Corresponding author. Current address: Shaanxi Key Laboratory of Agricultural and Environmental Microbiology, College of Life Sciences, Northwest A&F University, No. 22 Xinong Road, Yangling, Shaanxi Province, PR China, 712100.E-mail address: [lydiayliu@yahoo.com](mailto:lydiayliu@yahoo.com)

**
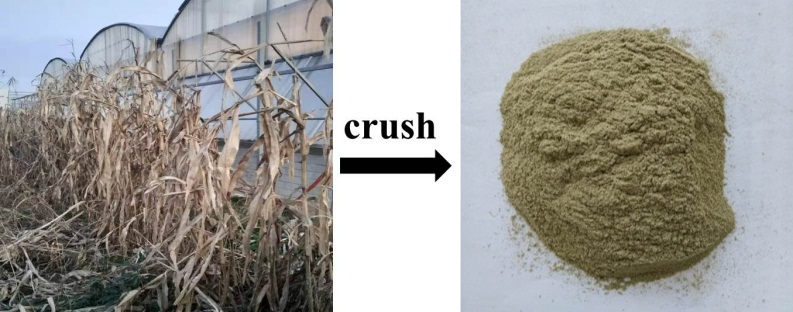
**

**Fig. S1** Corn stover (whole corn except corn ear) was crushed into less than 0.5 mm powder (passed through a 40 mesh screen).


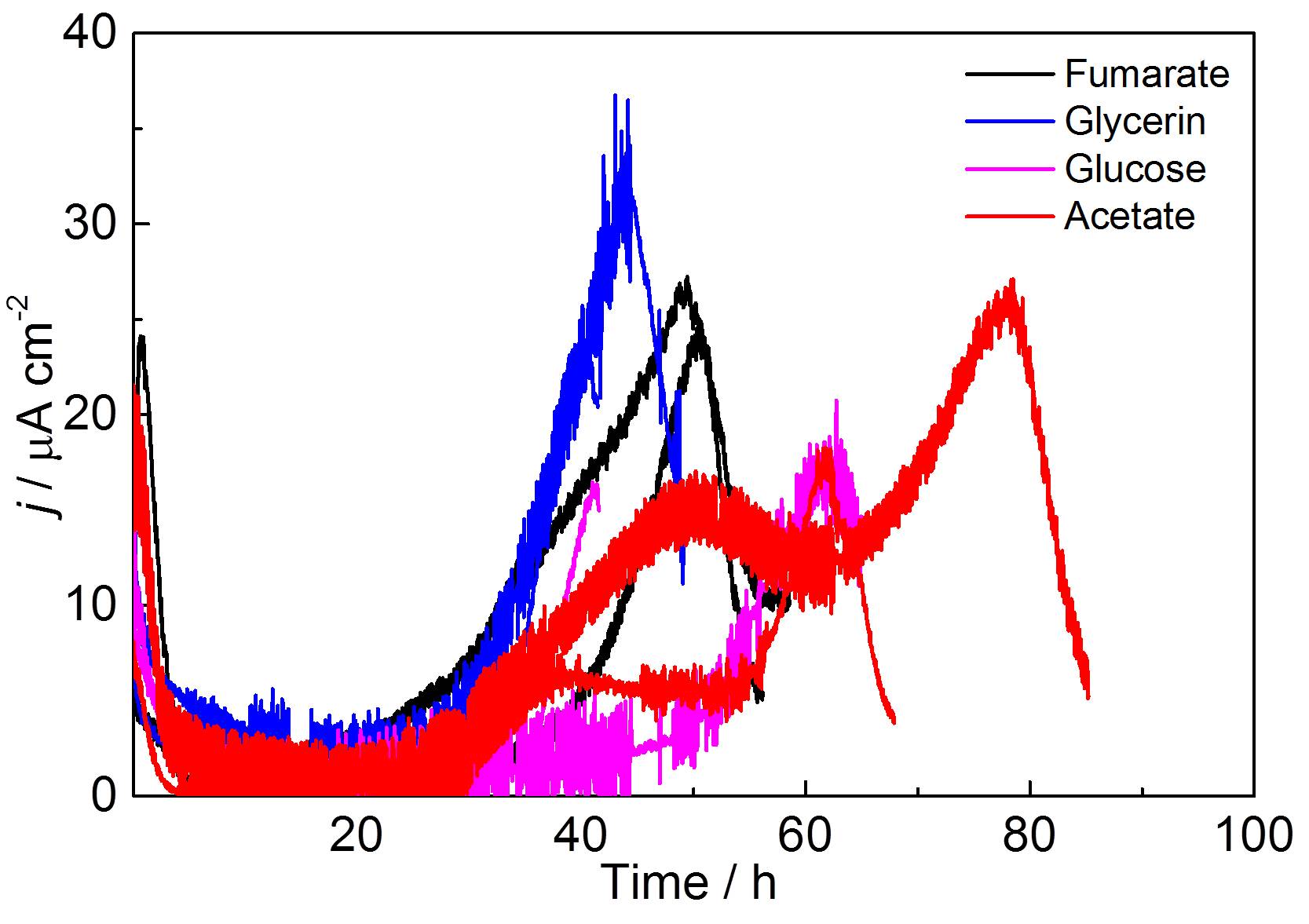


**Fig. S2** Chronoamperometric curves of the three-electrode system inoculated with strain Lsc-8 with different carbon sources at a constant potential of 0.35 V.


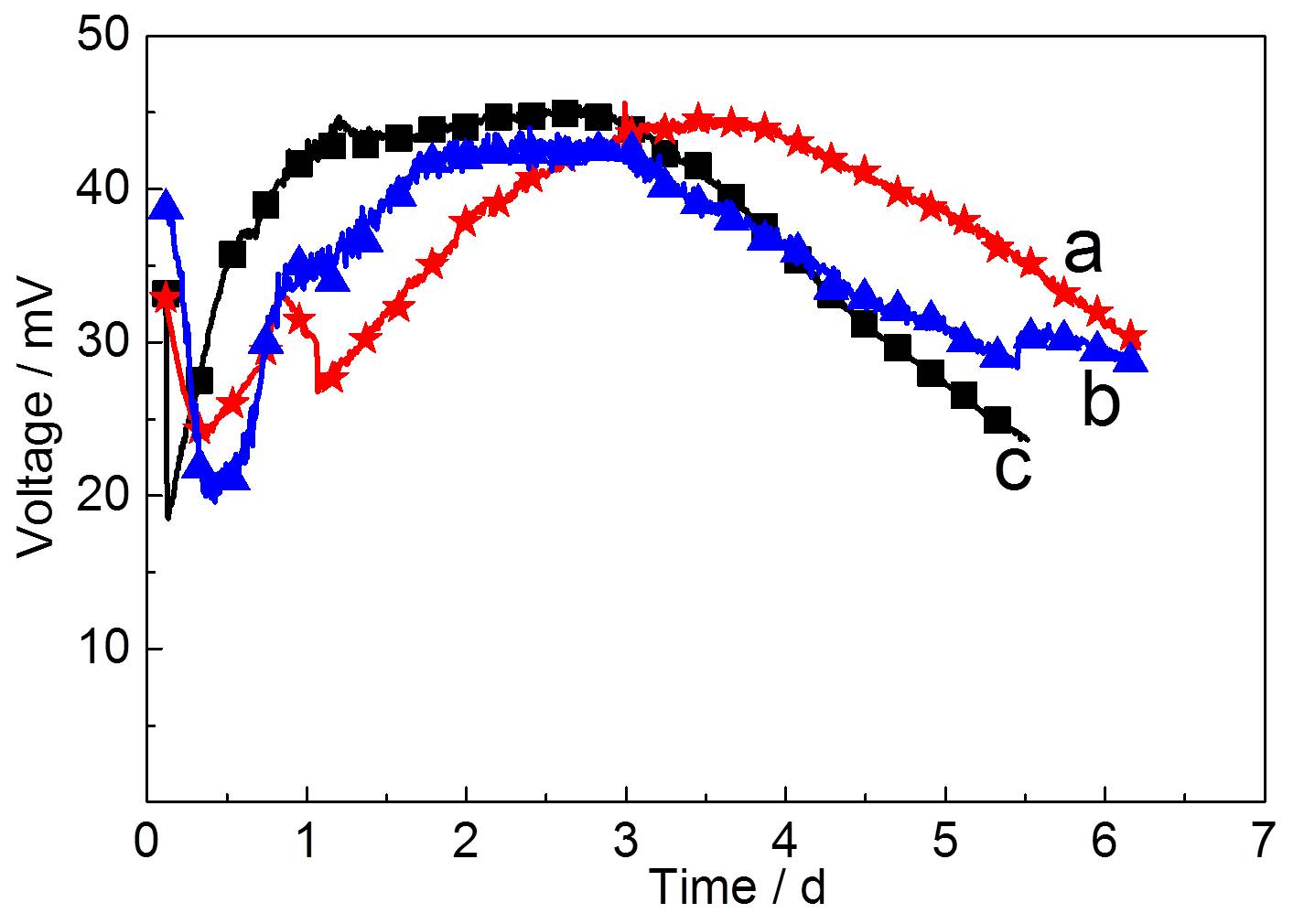


**Fig. S3** The output voltages of the MFCs inoculated with strain Lsc-8 with raw corn stover as the carbon source; curves a, b and c denote three replications.


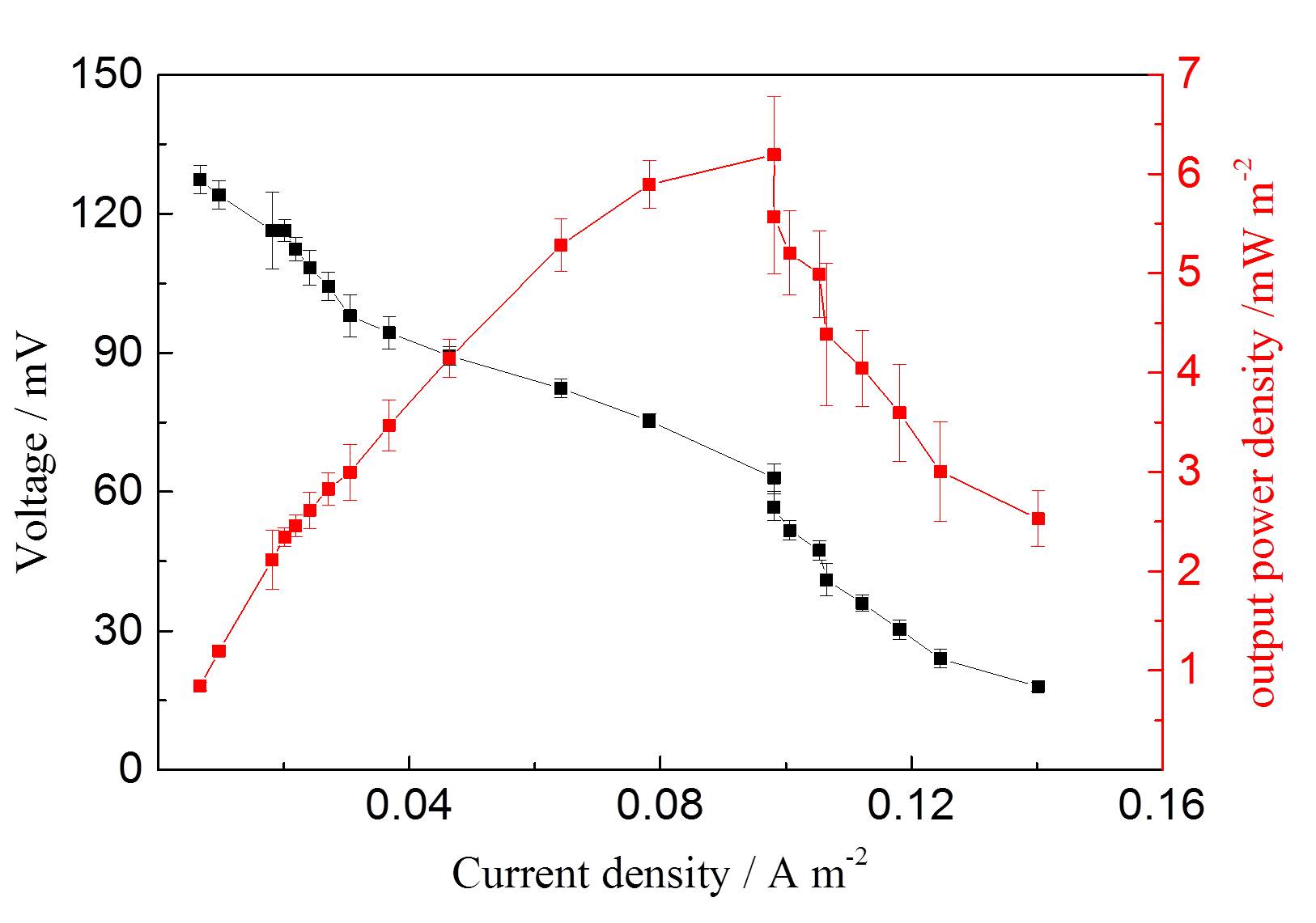


**Fig. S4** Curves of the output voltage and power density as a function of current density in MFCs inoculated with strain Lsc-8 with corn stover as the carbon source.


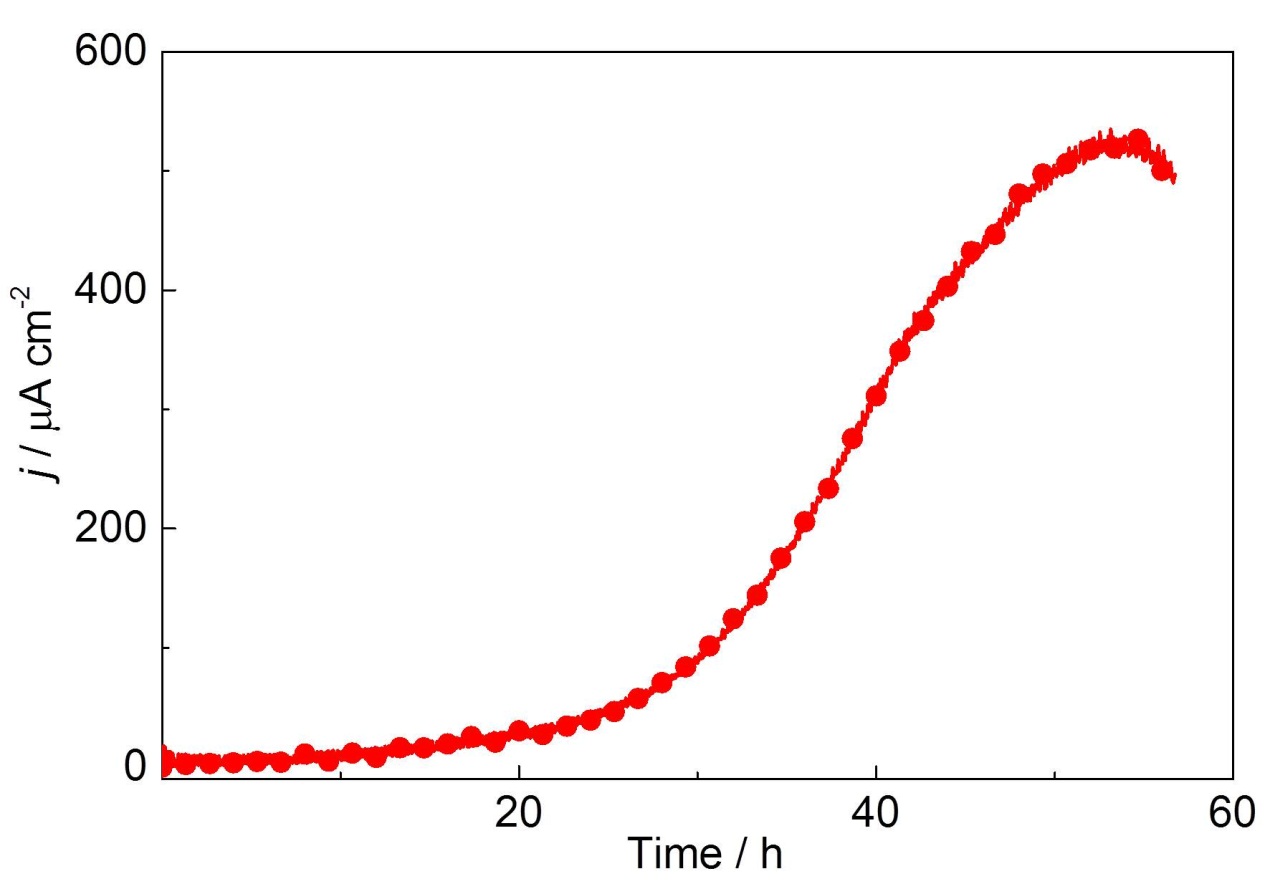


**Fig. S5** Chronoamperometric curves of the three-electrode system inoculated with *G. sulfurreducens* PCA with acetate as the carbon source at a constant potential of 0.3 V.


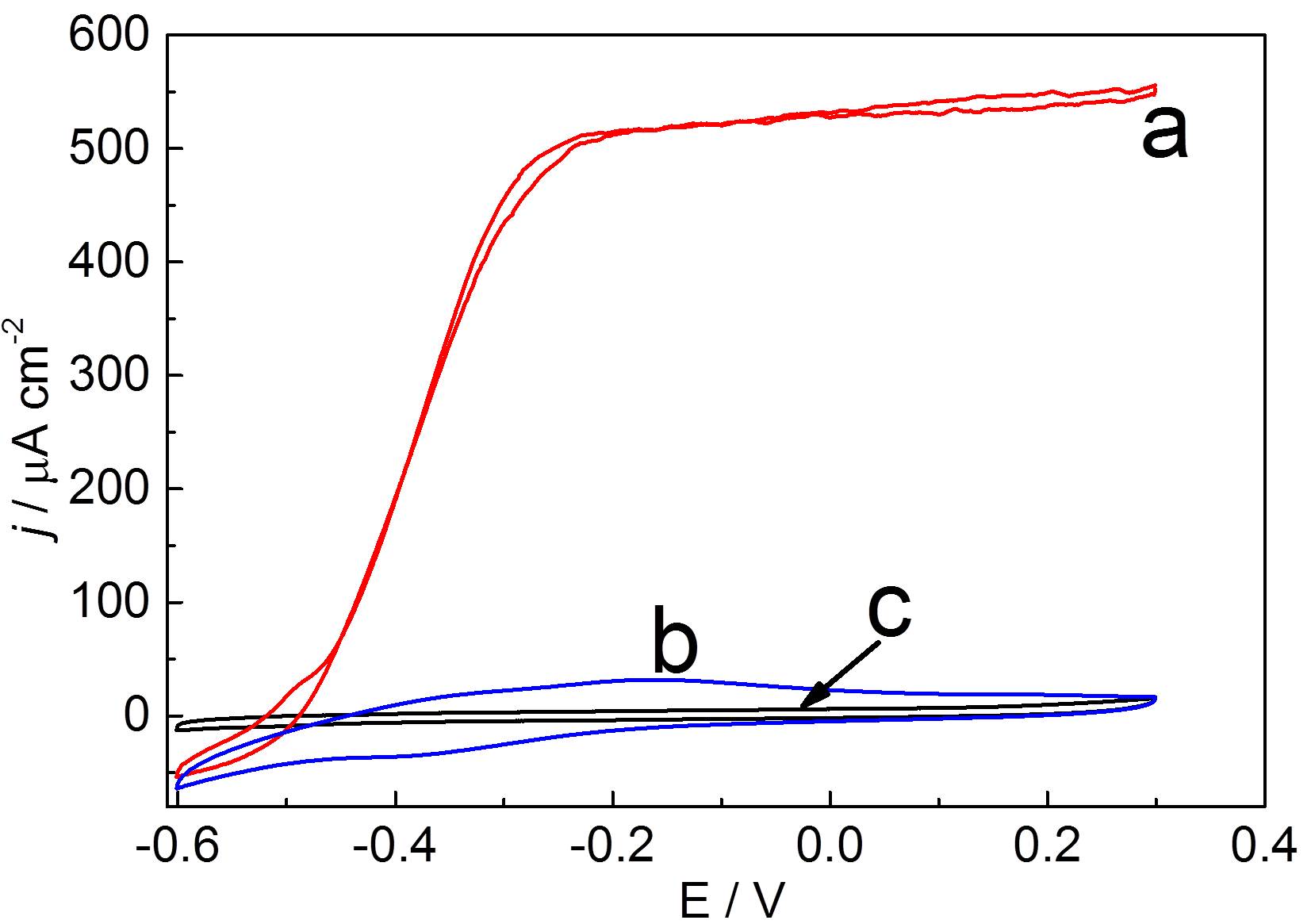


**Fig. S6** Cyclic voltammograms of the three-electrode inoculated co-culture system with CMC as the carbon source: (a) when the output current density reached its highest value, (b) when the output current density dropped to zero, (c) at the start of operation (the control).


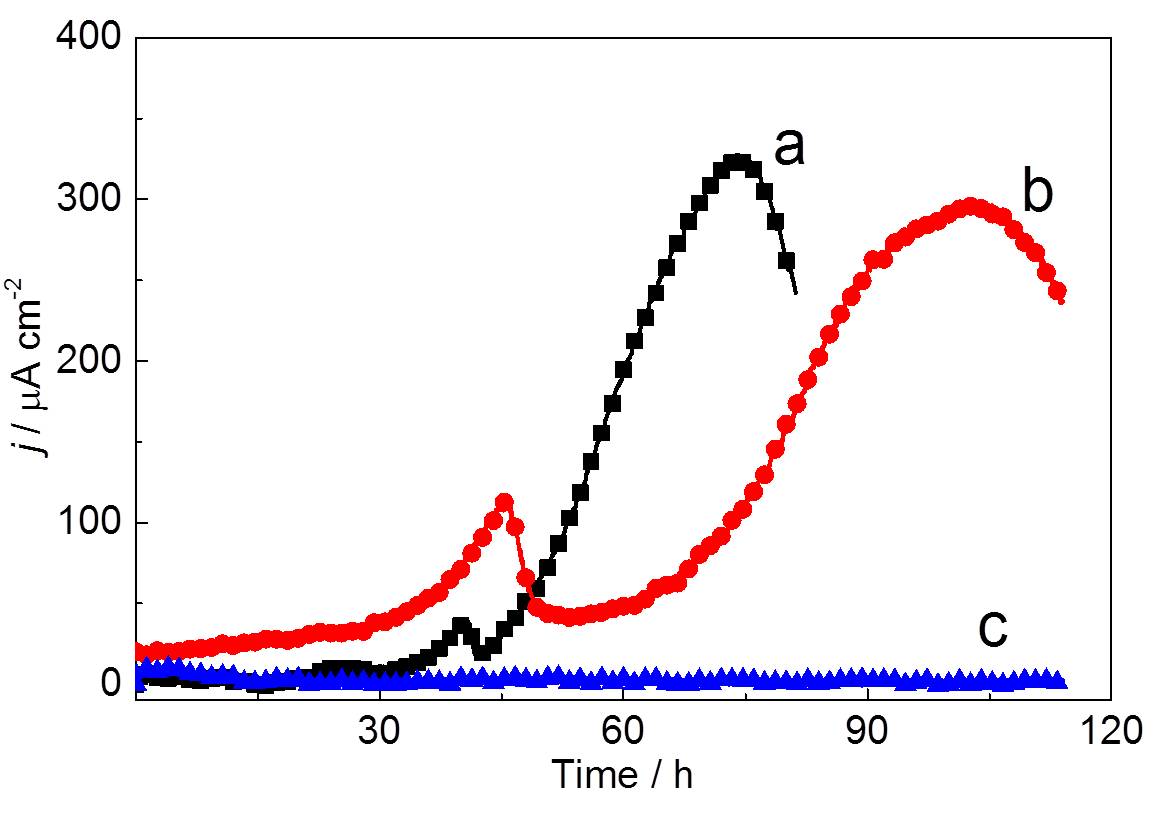


**Fig. S7** Chronoamperometric curves of the three-electrode inoculated co-culture system (curves a and b denote two replications) or *G. sulfurreducens* PCA (curve c) with glucose as the carbon source at a constant potential of 0.3 V.


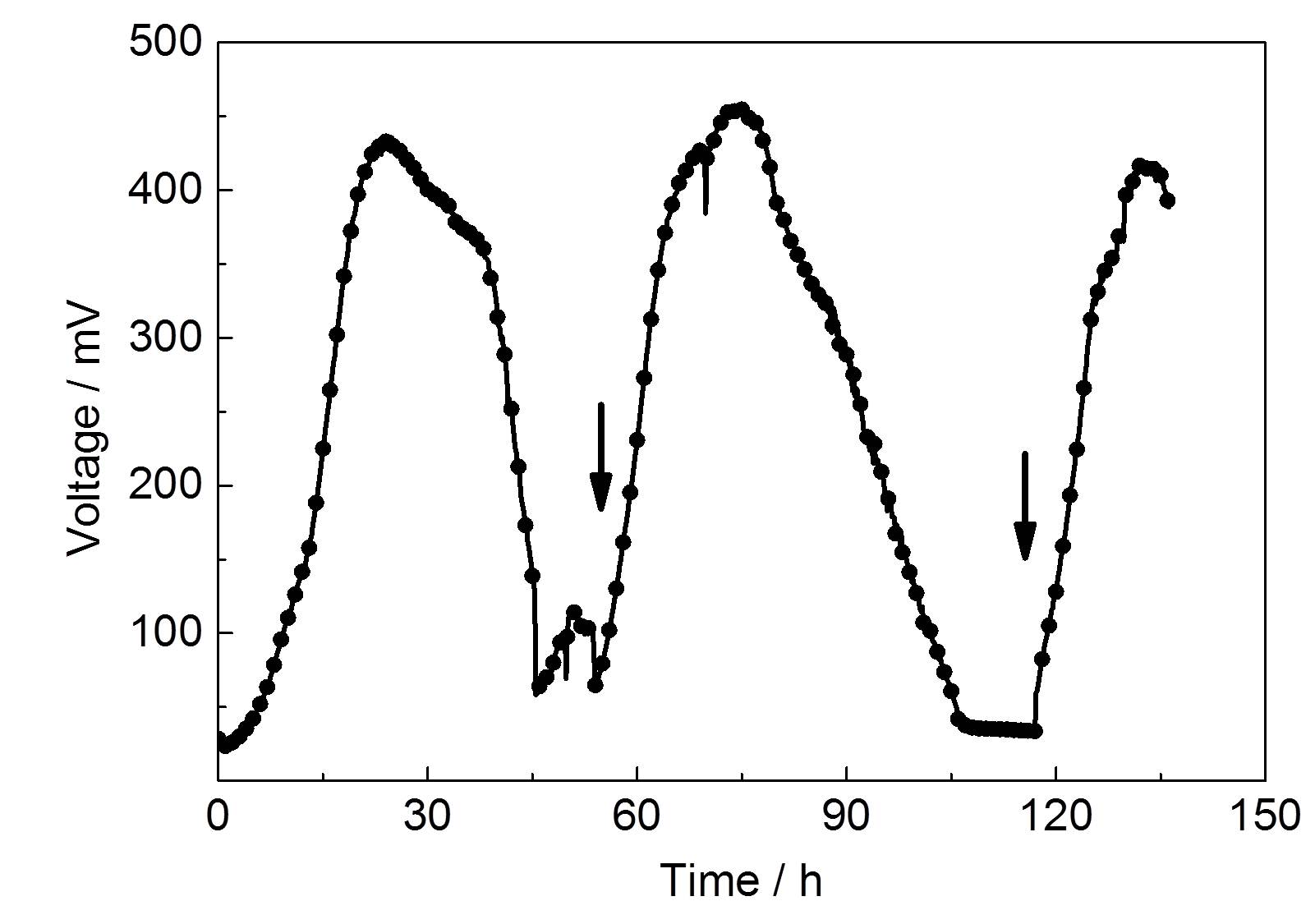


**Fig. S8** The output voltages of the MFCs-inoculated co-culture system with CMC as the carbon source. Arrows show replacement of the medium in the MFCs with new CMC medium.


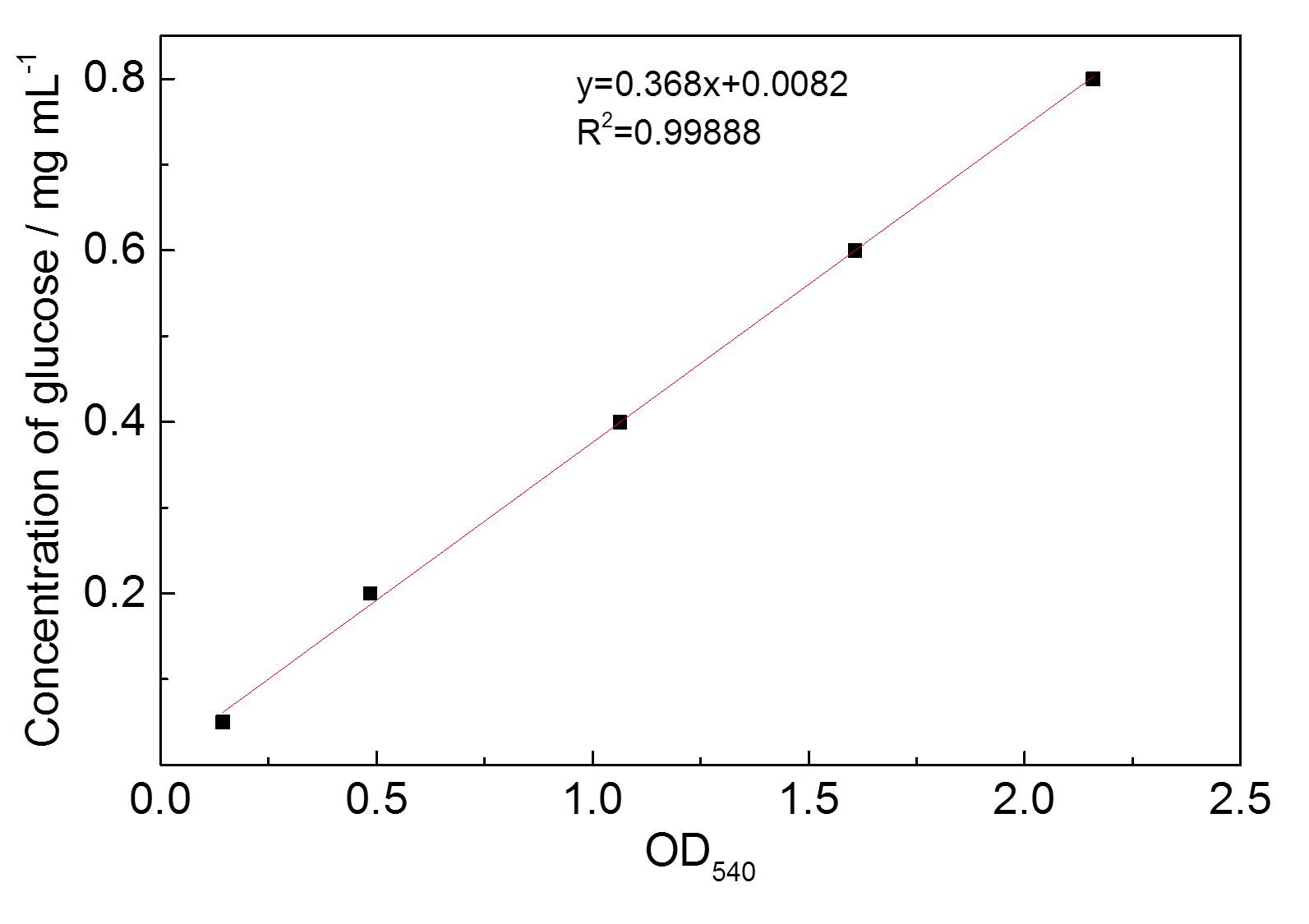


**Fig. S9** The standard curve of OD_540 nm_ versus glucose concentration (reducing sugar assay).

**Fig. S10** The standard curve of OD_620 nm_ versus glucose concentration (soluble total sugar assay).


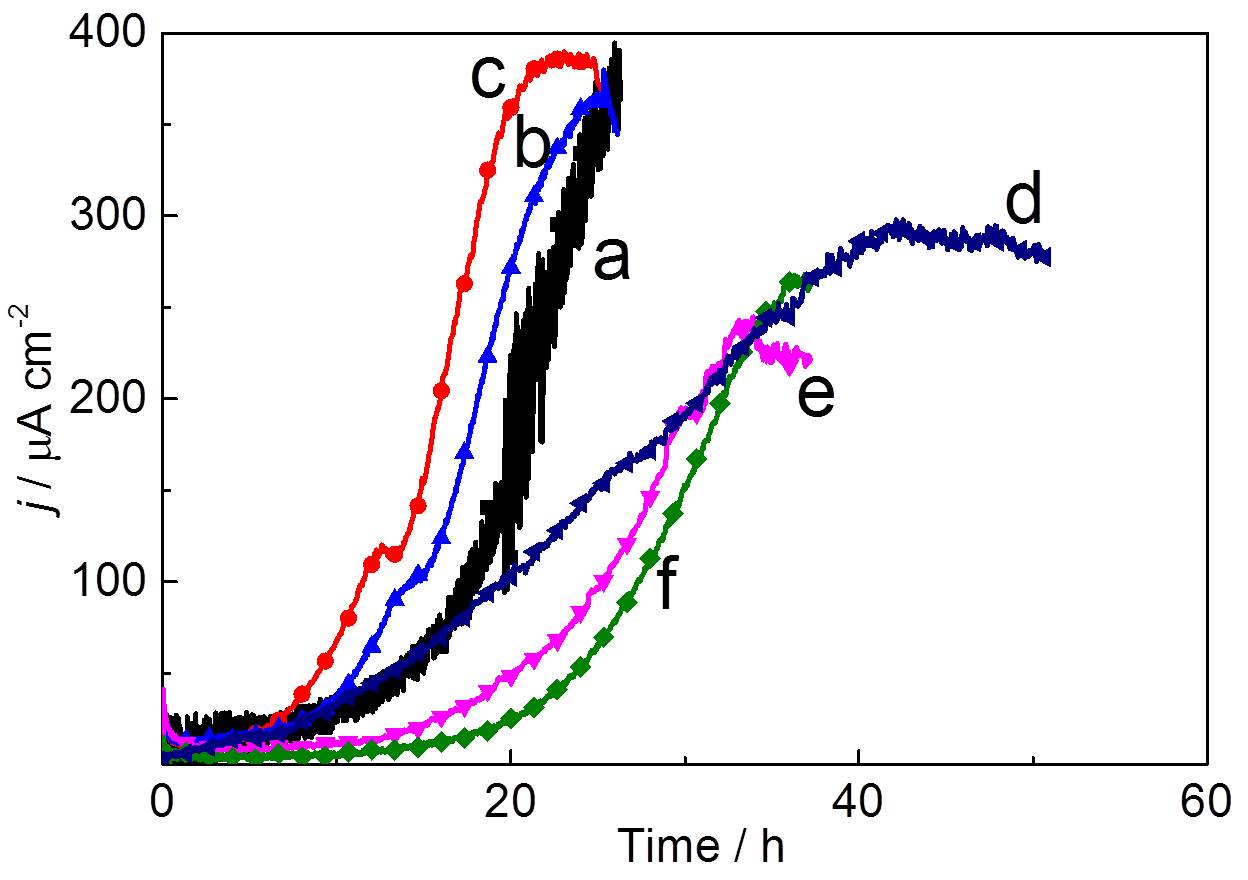


**Fig. S11** Chronoamperometric curves of the three-electrode inoculated co-culture system with medium ІІІ (curves d, e and f denote three replications) and fermentation broth of medium ІІІ (curves a, b and c denote three replications) as the carbon source.


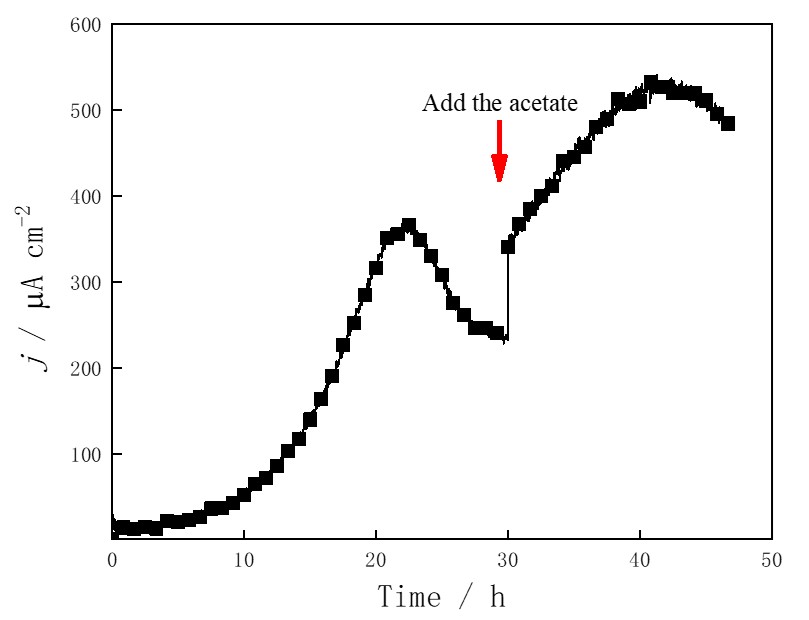


**Fig. S12** Chronoamperometric curves of the three-electrode inoculated co-culture system with fermentation broth of medium ІІІ as the carbon source. Arrows indicate the addition of acetate.


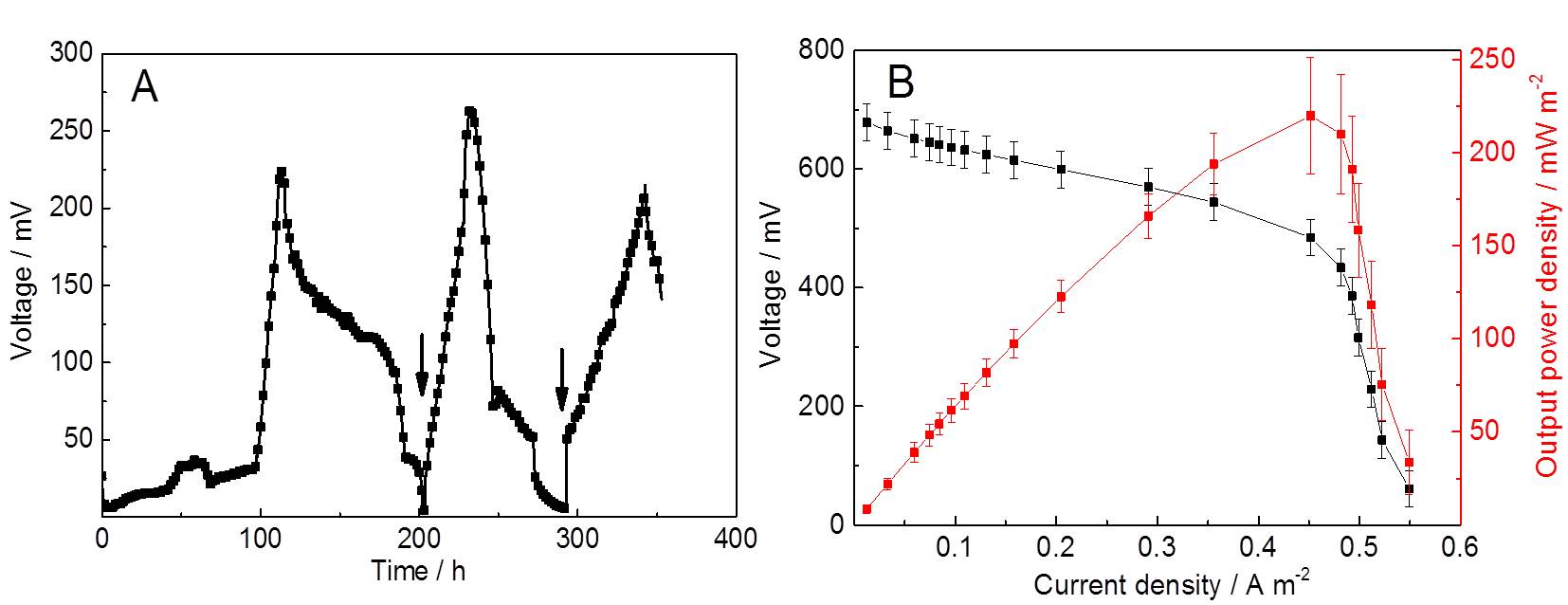


**Fig. S13** (A) The output voltages of the MFCs-inoculated co-culture system with fermentation broth of medium ІІІ as the carbon source. Arrows show replacement of the medium in the MFCs with the same carbon source. (B) Curves of the output voltage and power density as a function of current density in the MFCs-inoculated co-culture system with fermentation broth of medium ІІІ as the carbon source.


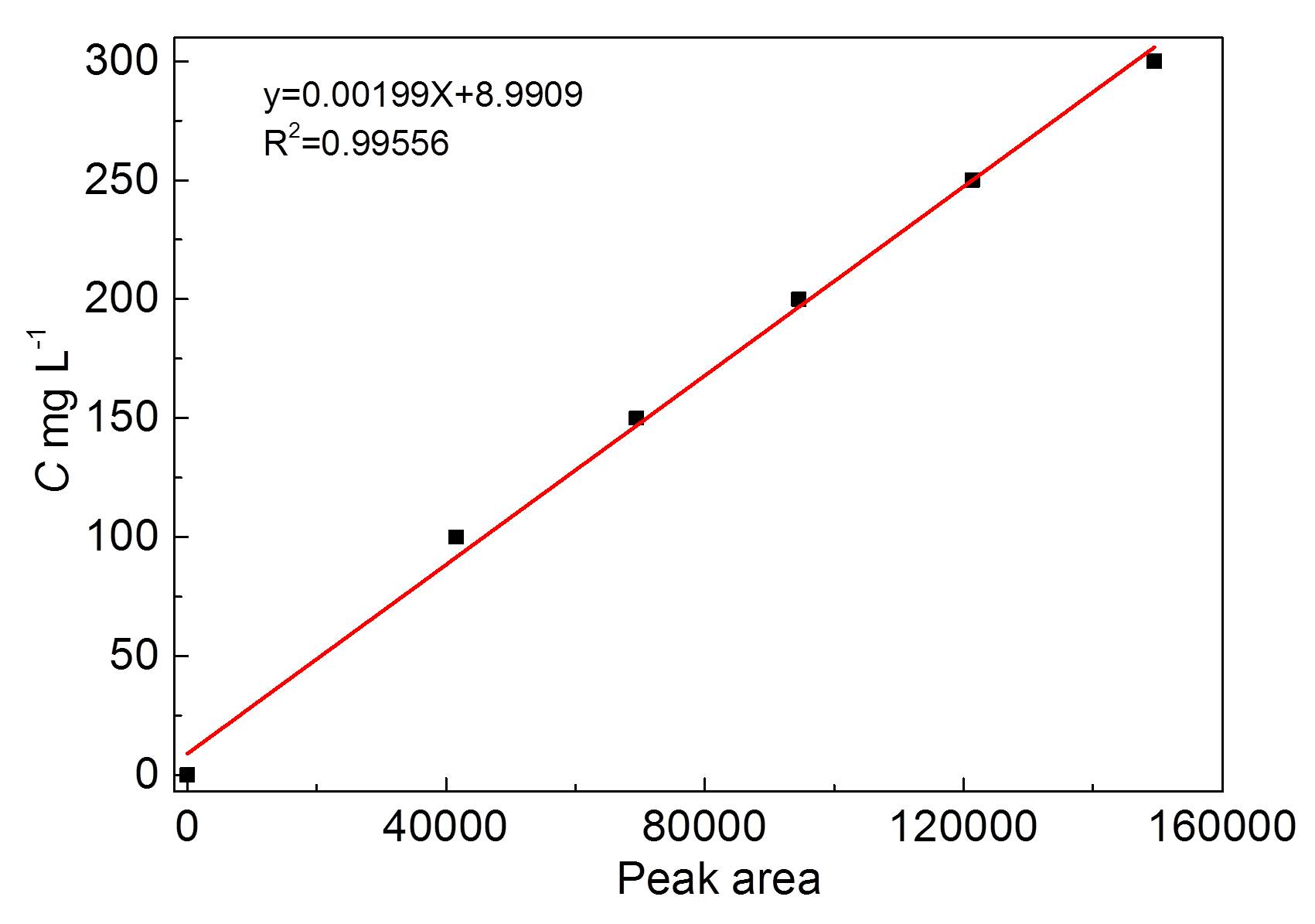


**Fig. S14** The standard curve of peak area versus acetic acid concentration.

**Table S1** Thecellulase activity of strain Lsc-8

| Strain | Cellulase Activity (U·mL^-1^) |
| --- | --- |
| Strain Lsc-8 | 0.20 ± 0.0071 |
